# Transactions of the Medical Society of the State of West Va.

**Published:** 1883-08-20

**Authors:** 


### *TRANSACTIONS OF THE MEDICAL SOCIETY OF THE STATE OF WEST VIRGINIA.*

HELD IN GRAFTON, MAY 1883.

A book of 79 oc. pages, containing Constitution and By-Laws, with Minutes of the Sixteentb Annual Session, with the following interesting papers:

Annual Address, by President B. W. Allen, M. D.

Report of Committee on Epidemic Diseases, by R. W. Hall, M.D.

Report of Committee on New Remedies, by J. M. Lazzell, M.D.

The Germ Theory of Disease, by E. C. Myers, M.D.

Abuse of Ergot in Obstetric Practice, by D. Porter, M.D.

Insanity as a Disease, and the Duty of the Medical Profession of the State Concerning its Management, by George H. Carpenter, M.D.

Puerperal Fever, with Special Reference to Treatment, by S. L. Jefferson, M.D.

Report of a Case of Intra-Peritoneal Hæmatocele, by R. W. Hall, M. D.

Record of some Anomalous Obstetrical Cases, by C. F. Ulrich, M.D.

A list of members, numbering 134, closes the volume. Some of the papers contain valuable suggestions, which we hope to notice, as time and opportunity present.

OFFICERS ELECT.

PRESIDENT—A. Gerstell.

VICE PRESIDENTS—C. T. Richardson, M.D.; A. F. Stifell, M.D.; D. Porter, M.D.

TREASURER—J. A. Campbell, M.D.

SECRETARY—S. D. Jepson, M.D.

Next place of meeting, Clarksburg, 3d Wednesday in May, 1881.

